# Decomposition Characteristics of Lignocellulosic Biomass in Subtropical Rhododendron Litters under Artificial Regulation

**DOI:** 10.3390/metabo13020279

**Published:** 2023-02-15

**Authors:** Puhang Zhang, Jian Lin, Jiangtao Hao, Chaochan Li, Wenxuan Quan

**Affiliations:** Guizhou Provincial Key Laboratory for Information Systems of Mountainous Areas and Protection of Ecological Environment, Guizhou Normal University, Guiyang 550001, China

**Keywords:** litter, controlled experiment, decomposition characteristics, lignin, cellulose

## Abstract

The nutrient turnover of subtropical rhododendron forests is slow, natural regeneration is difficult, and the decomposition of litter is slow. Lignin, cellulose, and hemicellulose are the key factors affecting the decomposition rate of litters. In this study, the litters of three forest stands, namely evergreen broadleaf *Rhododendron delavayi*, evergreen broadleaf *Rhododendron agastum*, and deciduous broadleaf mixed forest, were taken as the research objects to explore the dynamic changes and effects of lignin, cellulose, and hemicellulose contents in litters of different stands under indoor artificial control measures. Exogenous nitrogen, phosphorus, alkaline substances, and microbial agents were added to decompose litters in the laboratory for 140 days. Our results showed that (1) the contents of lignin and cellulose in the litters of the three stands decreased significantly in the early stage of decomposition and the content of hemicellulose was stable, and (2) low concentrations of nitrogen and phosphorus can accelerate the degradation of lignin, cellulose, and hemicellulose in litters of the three stands and thus promote the decomposition of litters. This study provides basic data for the nutrient return of artificial intervention in subtropical rhododendron forests in China.

## 1. Introduction

As an important component connecting forest vegetation and soil, the decomposition process of litter is an important link between nutrient cycling and energy flow in forest ecosystems [1,2,3]. Lignocellulosic biomass consists of hemicellulose, cellulose, and lignin. Lignocellulosic biomass is the most abundant and difficult component in litter decomposition and plays an important role in litter decomposition [4]. Compared with experiments conducted in forest habitats, the results of laboratory experiments are less affected by microenvironmental differences and extreme climates (such as snow and rain) and can reflect the decomposition characteristics of litters themselves relatively objectively [5,6]. However, previous studies on the degradation process of cellulose, lignin, and other refractory substances in litter were mostly carried out in forest habitats [7,8], and few studies have used indoor artificial control experiments to promote litter decomposition. Some studies conducted laboratory-simulated decomposition experiments on needle litters to test the effects of secondary metabolites on litter mass loss and C, N, and P release [9]. Other studies have used indoor laboratory simulations to create a series of microworlds, examining the toxicological effects of heavy metal pollution on microbial communities and functions associated with litter decomposition and the ability of artificial light to mitigate this phenomenon [10,11]. Therefore, when exploring the decomposition mechanism of litters, it is very important to clarify the characteristics of lignin, cellulose, and hemicellulose in litters through control experiments and artificial measures to promote decomposition. The response mechanism of litters to nitrogen addition in permafrost peatland in the Da Xing’anling Mountains, China, has been revealed [12].

Baili Rhododendron National Forest Park (abbreviated as Baili Rhododendron) is the largest subtropical evergreen rhododendron forest in the world and is located in southwest China. However, due to the number of tree species is relatively small, weak forest ecological stability, and natural regeneration obstacles, litter has become an important part of forest stand management [13]. Through field investigation and research, the team found that there was a thick litter and humus layer under the rhododendron forest enriched in decomposed substances, such as lignin and cellulose, and rich in organic acids and allelopathic substances, which affected the natural regeneration of the rhododendron forest to a certain extent [14]. In this study, three forest stands, *Rhododendron delavayi* forest, *Rhododendron agastum* forest, and mixed forest (mixed forests contain litter from *Rhododendron delavayi* and *Rhododendron agastum* in a ratio of approximately 1:1), were selected as the research objects. The litter decomposition method of the laboratory control treatment was adopted by setting up seven different treatments (nitrogen addition, phosphorus addition, alkali lime addition, three different microbial agents, and control group) to explore the effects of artificial control measures on the litter decomposition rate and release characteristics of lignin, cellulose, and hemicellulose in the three stands. We hypothesized that (1) one or more treatments can significantly change the content and release rate of lignin, cellulose, and hemicellulose in litters during decomposition, and (2) one or more treatments will accelerate the decomposition of litters. The purpose of this study is to understand the degradation of lignin, cellulose, and hemicellulose in rhododendron litter under different artificial control treatments, provide new ideas for litter degradation and artificial-assisted natural regeneration of rhododendron communities, and provide a reference for accelerating the decomposition of litter and promoting nutrient recycling in wild rhododendron forests.

## 2. Materials and Methods

### 2.1. Study Area and Sample Collection

The elevation of the Baili Rhododendron (27°10′07″–27°17′55″N, 105°50′16″–106°04′57″E) scenic spot is generally 1450–1800 m and the climate type belongs to the warm temperate humid monsoon climate. The average annual relative humidity is 84%, the average annual temperature is 11.8 °C, and the annual rainfall is 1150.4 mm. The average annual relative humidity is 84%, the average annual temperature is 11.5 °C, the annual average accumulated temperature is 420 °C [14,15], the pH value of soil is 4.2–5.1, and the soil type is histic (organic soils) [16]. The evergreen deciduous broad-leaved forest zone in the Baili Rhododendron Forest is the main established species and dominant species in the Baili Rhododendron Forest community.

In October 2021, undisturbed soil of 0–20 cm, humus, and fallen leaves of *Rhododendron delavayi*, *Rhododendron agastum*, and mixed forest (3 forest stands) were collected. The collected samples were divided into 10 equal parts after being taken back to the laboratory. The soil, humus, and leaf litter were placed in 30 high-density polyethylene (HDPE) plastic baskets (55 cm long, 43 cm wide, 19 cm high, with water permeable holes at the bottom) from bottom to top. The dry weight of soil and humus was 9.2 kg per sample, for a total of 30 samples. One leaf litter sample was placed on the surface of each plastic basket, and the dry weight of each basket was 0.8 kg.

### 2.2. Experimental Design

Sample collection and processing: experiments were conducted indoors to increase nitrogen (N) and phosphorus (P), apply alkali lime (acid and alkali substances), and apply 3 different microbial agents. The indoor experiment was conducted at Guizhou Normal University in Guiyang. The climate was subtropical humid and mild, with an average annual temperature of 15.3 °C and an average annual relative humidity of 77%. There is no severe cold in winter, and the coldest cold occurs in early January, with an average temperature of 4.6 °C. The plastic basket should be placed indoors with good ventilation, and the temperature changes are consistent with the ambient temperature (from November to April, the average maximum indoor temperature is 6 to 19 °C, and the average minimum indoor temperature is 1 to 10 °C). A control group (not added) was set up in the experiment. According to the local summer rainfall records for many years (2012–2022) and the background detection values of natural N and P deposition in this area, the intensity of simulated N and P addition was set [17]. The nitrogen addition level from low to high was set as 0 g·N·m^−2^·yr^−1^ (CK), 2 g·N·m^−2^·yr^−1^ (LN), and 4 g·N·m^−2^·yr^−1^ (HN). The nitrogen fertilizer was urea, which contains 46% nitrogen, and the corresponding amount of urea was dissolved in water for spraying. The phosphorus addition level from low to high was set as 0 g·P·m^−2^·yr^−1^ (CK), 5 g·P·m^−2^·yr^−1^ (LP), and 20 g·P·m^−2^·yr^−1^ (HP). The phosphate fertilizer used in the experiment was NaH_2_PO_4_, and the corresponding amount of NaH_2_PO_4_ was dissolved in water for spraying. Urea and NaH_2_PO_4_ are nitrogen fertilizer and phosphate fertilizer commonly used in aquaculture in China and are easily soluble in water. The addition level of soda lime (a mixture of NaOH and Ca(OH)_2_, pH: 12–14) was set as 0 g/m^2^ (CK), 100 g/m^2^ (LS), and 400 g/m^2^ (HS). Three kinds of microbial agents were selected: straw decomposing agent (JG), plant microbial agent (EM), and organic fertilizer fermentation agent (YJF). The straw decomposing agent is the product of Zhengzhou Wangnongbao Biotechnology Co., Ltd. (Zhengzhou, China), which mainly contains *Lactic acid bacteria*, *Saccharomyces*, and other complex bacteria. The EM bacteriological agent was selected from Henan Nongfukang Biotechnology Co., Ltd. (Henan, China), which mainly contains complex bacteria, such as *Bacillus licheniformis* and *Bacillus subtilis*. YJF is the product of Shandong Beijia Biotechnology Co., Ltd. (Shandong, China) and mainly contains *Actinomycetes*, *Trichoderma*, and other complex bacteria.

The litter of each stand was divided into 12 pieces on average, with 1 piece for each treatment. Exogenous substances were added once every 15 days, and the same amount of distilled water was applied to the control group (CK) and supplemented with water every week according to the average value of local precipitation in the Baili Rhododendron (humidity was controlled at approximately 60%). The experimental period was 140 days, from 29 November 2021 to 23 April 2022.

Sampling: the litter leaves were recovered once at 20, 50, 90, and 140 days after the addition, and were recovered a total of 4 times. Sampling was undertaken using a 5-point method, which takes samples from the corners and the middle of the plastic basket and mixes them together in a sealed bag (remove twigs, bark, and other debris). The fresh weight of each basket was approximately 30 g and the samples were stored in a refrigerator at 4 °C.

### 2.3. Analytical Methods and Calculations

The leaves in the sample were separated from dead branches, the visible mycelia on the surface were removed, and the leaf litter was dried to a constant weight. Then, the leaf litter was weighed by an electronic balance to calculate the mass loss rate and residual mass. The leaf litter was ground according to the measurement requirements, passed through a sieve with a diameter of 0.425 mm, placed in sealed bags, marked accordingly, and stored in a refrigerator at 4 °C before testing. Lignin, cellulose, and hemicellulose were determined using a biochemical kit from Suzhou Grisi Biotechnology Co., Ltd. (Suzhou, China) according to the manufacturer’s instructions. Sample preparation and detection were carried out in strict accordance with the steps in the instructions. During the test, the microplate reader was preheated for 30 min, and the temperature was set at 25 °C. Approximately 2 mg of the sifted and dried stem powder was added to 1.5 mL 80% ethanol for shaking and mixing, and then bathed in water at 50 °C for 20 min and centrifuged at 12,000 rpm for 10 min. The sediments were used for the determination of lignin, cellulose, and hemicellulose contents. Lignin was determined by the acetylation method [18]. The phenolic hydroxyl group in lignin was acetylated, and its characteristic absorption peak was at 280 nm. The light absorption value at 280 nm was positively correlated with the lignin content [19]. Cellulose was processed by anthrone colorimetry [20]. After adding concentrated sulfuric acid, anthrone reagent was used to react with furfural compounds to generate a blue-green substance. The substance had a maximum absorption peak at 620 nm by spectral scanning and the cellulose content was obtained. Hemicellulose was determined by the acid heating-colorimetric method [18]. Under acidic conditions, it was hydrolyzed into xylose by heating. The content of xylose generated was detected by the colorimetric method, and then the hemicellulose content was calculated. Due to the mature technology of the company’s kit, the values read under the same sample differ little, so no duplicate samples were set during testing. The microplate reader used was a SPECTRAMAX PLUS384 continuous wavelength microplate reader (Molecular Devices, Sunnyvale, CA, USA).

Litter residual rate calculation [21]:

(1) $$R=\left(\frac{M_{t}}{M_{0}}\right)\times 100\%$$

where *R* represents the litter residual rate; *M_t_* represents the dry mass of litter samples at sampling time *t*, g; and *M*_0_ represents the initial dry mass of litter without decomposition, g.

Calculation of litter decomposition rate [22]:

(2) $$L=\left(1-\frac{M_{t}}{M_{0}}\right)\times 100\%$$

where *L* stands for the litter decomposition rate; *M_t_* represents the dry mass of litter samples at sampling time *t*, g; and *M*_0_ represents the initial dry mass of litter without decomposition, g.

Determination of lignin (*L*), cellulose (*C*), and hemicellulose (*H*) [23,24]:

(3) $$L\left(\%\right)=\left\{\frac{\left(\Delta A+0.003\right)}{10.615}\times \frac{V_{1}}{W}\times D\times 0.1\right\}\%=\left\{0.1413\times \frac{\left(\Delta A+0.003\right)}{W}\times D\times 0.1\right\}\%$$

where ∆*A* = *A* measurement tube − *A* blank tube, *V*_1_: total volume after constant volume, 1 mL; *W*: sample weight, 1.5 mg = 1.5 × 10^−3^ g; *D*: dilution ratio.

(4) $$C\left(\%\right)=\left\{\frac{\left(\Delta A-0.0033\right)\times V_{1}}{9.9647\times W\times \frac{V_{1}}{V}}\times 0.9\times D\times 10^{-3}\times 100\right\}\%=\left\{0.0524\times \frac{\left(\Delta A-0.0033\right)}{W}\times D\right\}\%$$

where *V*: volume of added extract, 5.8 mL; *V*_1_: add sample volume, 0.125 mL; *W*: sampling mass, 0.02 g; *D*: dilution ratio; 0.9: conversion factor of glucose condensation into cellulose.

(5) $$\begin{array}{c} H\left(\%\right)=\left\{\frac{\left(C\;standard\times V_{1}\right)\times \Delta A}{A\;standard-A\;blank\times \left(\frac{W\times V_{1}}{V}\right)}\times 0.9\times D\times 10^{-3}\times 100\right\}\%\\ =\left\{0.261\times \frac{\Delta A}{W\left(A\;standard-A\;blank\right)}\times D\right\}\% \end{array}$$

where *C* standard: 0.5 mg/mL; *V*: added extraction liquid volume, 5.8 mL; *V*_1_: added sample volume, 0.06 mL; *W*: sampling mass, 0.02 g; 0.9: conversion factor of condensed hemicellulose; *D*: dilution ratio.

Lignin and cellulose release rate calculation [25]:

(6) $$E=\left[\frac{\left(C_{0}\times M_{0}-C_{t}\times M_{t}\right)}{C_{0}\times M_{0}}\right]\times 100\%$$

where *E* represents the release rate of lignin and cellulose; *C*_0_ represents the initial lignin and cellulose content of litter before decomposition, mg/g; *C_t_* represents the content of lignin and cellulose of litter during decomposition *t*, mg/g; *M*_0_ represents the initial dry mass of litter without decomposition, g; and *M_t_* represents the dry mass of litter at decomposition t, g. When *E* is positive, it shows net release, and when *E* is negative, it shows net enrichment.

### 2.4. Statistical Analysis

R 4.1.0, SPSS 19.0, and Origin Pro 2022 software were used for data analysis. The significant differences in the degradation of lignin, cellulose, and hemicellulose, mass decomposition rate of litters, and release rate of lignin and cellulose by different artificial treatments of different rhododendron litters were all analyzed by 2-way ANOVA to investigate the mass of litters and the degradation rate of lignin, cellulose, and hemicellulose in 3 stands during decomposition times. The initial lignin, cellulose, and hemicellulose contents of the 3 litters were analyzed by 1-way ANOVA, and the results of variance analysis were significant (*p* < 0.05). The Duncan’s significant difference test was used to determine the difference between the means.

## 3. Results

### 3.1. Initial Characteristics of Lignin, Cellulose, and Hemicellulose in Litter of Three Forest Stands

The initial contents of lignin, cellulose, and hemicellulose in the litters of *Rhododendron delavayi*, *Rhododendron agastum,* and the mixed forest are shown in Table 1. The average initial contents of lignin, cellulose, and hemicellulose in the three litters were 119.31 mg/g, 10.44%, and 3.89%, respectively. There were some differences in the initial lignin, cellulose, and hemicellulose contents of the litters of the three rhododendrons, which might be due to the different enzyme activities and the effects of microorganisms on the decomposing enzymes in the litter decomposition process [26]. From high to low, the initial contents of lignin in the three litters were *Rhododendron agastum*, mixed forest, and *Rhododendron delavayi*. The initial lignin and cellulose contents in the litters of *Rhododendron delavayi* and *Rhododendron agastum* were nearly four times higher than the initial hemicellulose contents, while the initial lignin contents in the litters of the mixed forest were the highest, approximately two times and three times the initial cellulose and hemicellulose contents, respectively. There were significant differences in the initial cellulose content among the three forest stands (*p* < 0.05).

### 3.2. Characteristics of the Final Decomposition Rate of the Three Stands of Litter

**Figure 1:**
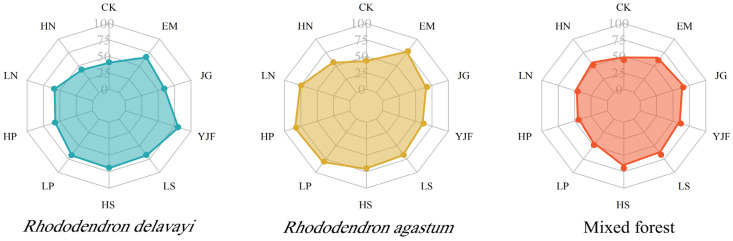
Final litter decomposition rate of the three stands after 140 days. (CK: control group; EM: plant microbial agent; JG: straw decomposing agent; YJF: organic fertilizer fermentation agent; LS: low concentration alkali lime; HS: high concentration alkali lime; LP: low concentration phosphorus; HP: high concentration phosphorus; LN: low concentration nitrogen; HN: high concentration nitrogen.).

The cumulative loss rate of litter in the three stands after 140 days of decomposition is shown in Figure 1. From high to low, the cumulative loss rates of the three litter control groups were 44.40%, 43.48%, and 41.02% in the mixed forest, *Rhododendron agastum*, and *Rhododendron delavayi*, respectively. Under the YJF treatment, the decomposition rate of *Rhododendron delavayi* litter was the highest, at 80.25%. The decomposition rates of the litter under LN, HP, LP, and EM were significantly higher than those under the other treatments, which were 74.62%, 82.22%, 79.72%, and 77.27%, respectively. The decomposition rate under the HP treatment was the highest at 82.22%. The decomposition rate of litter in the mixed forest was generally low, especially under the HP treatment, and the decomposition rate was the lowest at only 31.51%. The decomposition rate of litter in the *Rhododendron agastum* forest was relatively high in general, and the decomposition rate of leaf litter in the mixed forest was lower than that in the *Rhododendron delavayi* and *Rhododendron agastum*.

### 3.3. Dynamic Changes in Lignin, Cellulose, and Hemicellulose in the Decomposition Process of Litters

**Figure 2:**
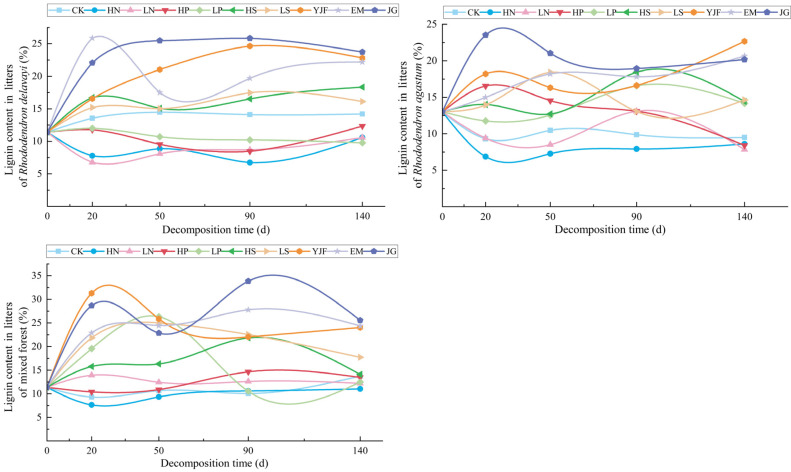
The variation in lignin content in litters of three stands under different treatments.

The variation in lignin content in the litter of the three stands under each treatment is shown in Figure 2, showing obvious fluctuations. The lignin concentration of litter under different forest stands and different treatments varied with decomposition time. The lignin content of the litters of the *Rhododendron delavayi* forest under nitrogen and phosphorus addition treatments was always lower than CK and initial values during the decomposition period of 0–140 days, and the value fluctuated the most under EM treatment, reaching the highest value at approximately 20 days after decomposition and then decreasing gradually at approximately 50 days after decomposition. The lignin content of the litters of the *Rhododendron agastum* forest under nitrogen addition and HP treatment was eventually lower than that of CK and the initial value, and the value of lignin under HP treatment gradually decreased approximately 90 days after decomposition and then lower than that of CK. The lignin content fluctuated the most under the JG treatment, reached a maximum during 20–50 days of decomposition, and slightly decreased after 50 days of decomposition. The lignin content of litters in the mixed forest was lower than that in CK under nitrogen and phosphorus addition treatments. The lignin content fluctuated the most under the JG and LP treatments. The lignin content in the litters of the mixed forest reached the highest value at approximately 20 days and 90 days after decomposition under the JG treatment and reached the highest value at approximately 50 days after decomposition of LP.

**Figure 3:**
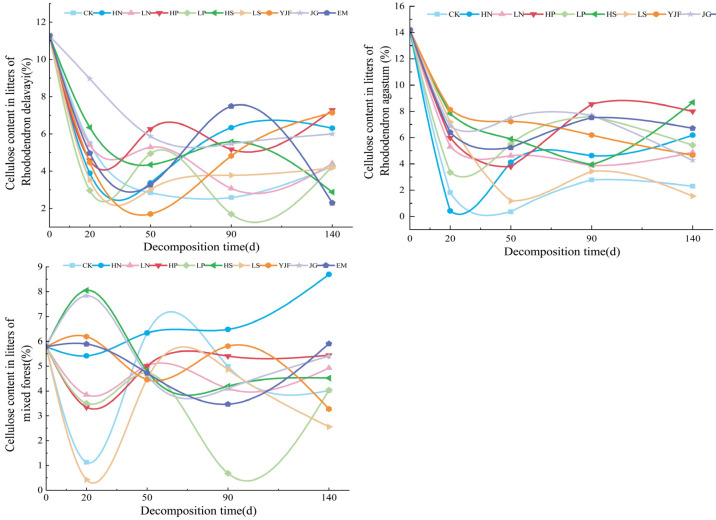
The variation in cellulose content in litters of three stands under different treatments.

In the decomposition process of litter, the cellulose changed, as shown in Figure 3, with irregular fluctuations. The cellulose content of litterfall of *Rhododendron delavayi* and *Rhododendron agastum* decreased rapidly from 0 to 20 days (early stage of the experiment) of decomposition and was significantly lower than the initial content after 140 days of decomposition, showing a trend of decreasing first and then increasing. The cellulose content of *Rhododendron delavayi* litter under LN, LP, LS, and EM treatments was ultimately lower than CK, while the cellulose content of *Rhododendron agastum* litter under nitrogen addition, LP, LS, YJF, and EM treatments was ultimately lower than CK. Cellulose concentration in litters of the mixed forest showed obvious irregular fluctuations, and the final cellulose concentration after 140 days of decomposition had little change compared with the initial concentration. Only the cellulose content under the LN, LP, and alkali lime treatments was lower than that under CK. The value under the HN treatment continued to increase during 0 to 140 days of decomposition, and the HS, LP, and LS treatments fluctuated greatly. At 20 days of decomposition, it increases and decreases significantly, respectively.

**Figure 4:**
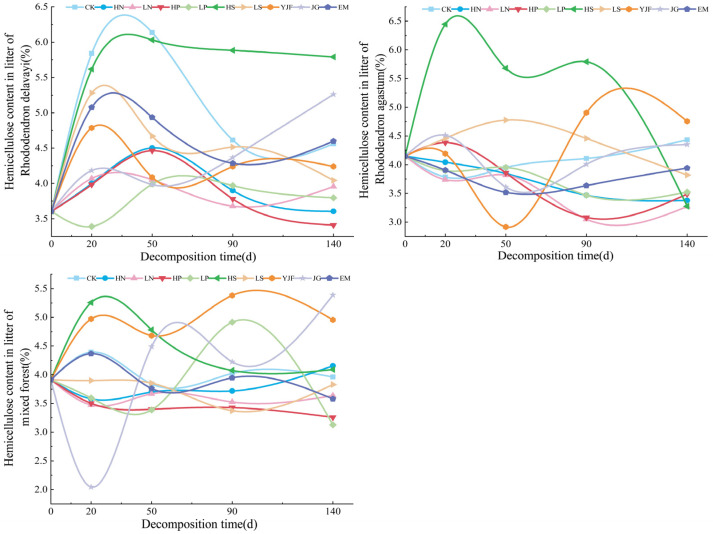
The variation in hemicellulose content in litters of three stands under different treatments.

In the decomposition process of litter, the hemicellulose changes, as shown in Figure 4, show a small fluctuation range overall. The hemicellulose concentration of all the litters did not vary by more than 2.2%. The hemicellulose content of the litters of *Rhododendron delavayi* under all treatments increased first and then changed gently. At the end of the decomposition experiment, except for HS, JG, and EM treatments, hemicellulose content was lower than in CK under other treatments, and the increase in alkali lime addition and CK decomposition was the largest at approximately 20 days. The fluctuation range of the litter of *Rhododendron agastum* was the largest under HS and YJF treatment, and the hemicellulose content under other treatments (except YJF treatment) was lower than in CK. The value of litters in the mixed forest fluctuated the most under YJF and JG treatments, and the hemicellulose content under JG treatment decreased to the minimum approximately 20 days after decomposition, while the hemicellulose content under LN, phosphorus addition, LS, and EM treatments was lower than in CK.

Table 2 shows the comparison of the effects of forest stands and treatments on the final lignin, cellulose, and hemicellulose contents. For the variables of forest stands and treatment methods, the analysis of F test results showed that the significance *p* < 0.001, showing a significance at the significance level of 1% and had a significant effect on the final contents of lignin, cellulose, and hemicellulose, all of which had a major effect. The interaction item stand × treatment mode had a significant effect on the final contents of lignin, cellulose, and hemicellulose, and there were interaction effects (*p* < 0.001).

### 3.4. Final Release Characteristics of Lignin, Cellulose, and Hemicellulose from Litters

**Figure 5:**
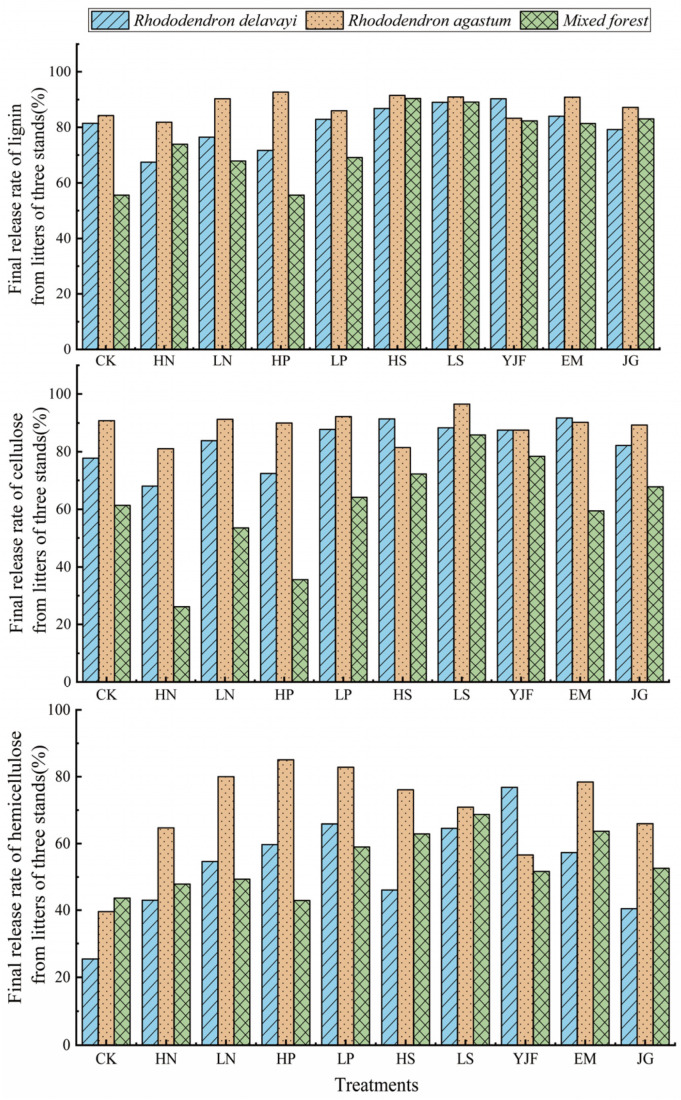
Final litter release rates of lignin, cellulose, and hemicellulose of the three stands.

After 140 days of the decomposition experiment, the final release rates of lignin, cellulose, and hemicellulose from the litter of the three stands are shown in Figure 5. In terms of the final release rate of lignin from litters, the values of the CK group of *Rhododendron delavayi*, *Rhododendron agastum*, and the mixed forest litter were 26.64%, 58.75%, and 32.87%, respectively. The lignin release rate of *Rhododendron delavayi* litter under the JG treatment was lower than that under CK, and the lignin release rate under the other treatments was higher than that under CK, among which the values of LN, LP, and YJF were more than 60%, which were 61.68%, 72.16%, and 60.52%, respectively. In addition, the lignin release rate of *Rhododendron agastum* litter under the YJF and JG treatments was lower than that under CK, and the lignin release rate under the other treatments was higher than that under CK. The values of the HN, LN, HP, and LP treatments reached more than 70%, which were 71.37%, 84.72%, 88.50%, and 77.91%, respectively. The litter values of mixed forest under HN, LN, LP, HS, and LS were higher than those under CK, which were 52.44%, 41.43, 43.68%, 55.98%, and 50.15%, respectively.

From the final release rate of cellulose in litters, the values of the CK group of *Rhododendron delavayi*, *Rhododendron agastum*, and mixed forest litters were 69.54%, 69.92%, and 61.33%, respectively. The final cellulose release rate of *Rhododendron delavayi* litter was higher than that of CK except for the HN treatment, and the values of the LN, LP, YJF, and JG treatments were higher than 85%. The values of the litter of Rhododendron agastum were higher than CK under all treatments, and the release rate of cellulose under LN, LP, and the three microbial agents was higher than 90%. Except for HN and HP, the cellulose release rate of litters in the mixed forest was higher than CK under the other treatments, and the values of LN, LP, YJF, and EM were higher than 75%.

From the final release rate of hemicellulose in litters, the values of *Rhododendron delavayi*, *Rhododendron agastum*, and mixed forest were 25.38%, 39.62%, and 43.67%, respectively, and the values under treatments were higher than CK. The value of *Rhododendron delavayi* litter under the LP, YJF, and EM treatments was more than 60%. The final release rate of hemicellulose from *Rhododendron agastum* litter was higher than 75% under the LN, HP, LP, and HS treatments. The value of mixed forest litter under the HS, LS, YJF, and JG treatments was higher than 60%. The release rates of lignin, cellulose, and hemicellulose in the three forest stands were different.

## 4. Discussion

### 4.1. Characteristics of Decomposition Rates of Forest Litter

Litter decomposition is an important link in nutrient cycling and carbon transformation in terrestrial ecosystems [27,28]. Lignin is an important organic component in plant litter and is generally considered to be highly limiting to the decomposition rate of litter [29]. In contrast, cellulose degrades faster during the decomposition of litter and plays a dominant role in the early stage of decomposition [30]. Relevant studies have found that lignin can physically protect cellulose from enzymatic hydrolysis, so cellulose cannot be degraded independently of lignin [31].

The decomposition of litters is affected by the mass of litters, the microbial community, and other external factors, and the change in temperature may lead to a change in the decomposition process of different forest stands of litters [12,32]. This experiment was carried out under the control of the laboratory. The decomposition environment of each litter was similar, reducing the effect of climate on litter decomposition, which mainly characterized the decomposition rate of the litter itself over time. In the decomposition process, lignin and cellulose were released quickly in the early stage and fluctuated to different degrees in the later stage, and the release rate tended to be slow.

The decomposition rate of litters in a single forest is higher than that in a mixed forest, which may be influenced by the difference in plant species combinations [33]. HN addition had little effect on the decomposition rate of *Rhododendron delavayi* litter, while LP addition had the best promotion effect. In this study, litters were not in direct contact with soil, and soil microorganisms had little influence on them. Therefore, the flora in microbial agents plays a decisive role in their decomposition process. It has been found that when the microbial biomass increases during the decomposition process of litters, it can promote the degradation of cellulose [34]. The promotion effects of the three microbial agents on the decomposition process were in descending order: YJF, EM, and JG. The addition of N and P to different degrees in the litter of *Rhododendron agastum* significantly promoted the decomposition of litter, HP > LP > LN > HN, and phosphorus addition was higher than nitrogen addition. Among the three microbial agents, EM had the best promoting effect followed by JG, and YJF had the weakest effect. The addition of HP to the litter of the mixed forest inhibited its decomposition, while the addition of N and P had little effect on its decomposition. Among the three microbial agents, JG > EM > YJF promoted decomposition. The addition of alkali lime to the litter of the three stands had an overall promoting effect, but there was little difference in the decomposition rate between the two levels. This result may be related to the concentration of Ca in the decomposition environment of litter. The higher the concentration of Ca, the higher the decomposition rate [35].

### 4.2. Variation Characteristics of Lignin, Cellulose, and Hemicellulose Contents in Litter

The content change rate of lignin and cellulose is large in the early stage of decomposition and gradually slows down in the middle and late stages. The contents of lignin and cellulose decreased rapidly in the early decomposition stage of litter. At this stage, more carbon sources easily decomposed, which was conducive to the degradation of lignin and cellulose. With the decomposition of litters, available nutrients decreased and the degradation of lignin and cellulose slowed [31]. Cellulose is more biodegradable than lignin and can be degraded by a variety of bacteria and fungi [30].

The concentrations of lignin and cellulose in the three litters treated with nitrogen decreased rapidly in the early stage of decomposition (approximately 0–20 d). A previous study showed that nitrogen addition could inhibit the lignin content in the early growth stage of bamboo forests [24]. In conclusion, nitrogen addition may affect the activity of some enzymes in litters, thus changing the content of metabolites. The three microbial agents rich in lactic acid bacteria, Bacillus subtilis, Actinomycetes, and other complex bacteria belong to bacteria, and the richness of these bacterial communities did not reach its peak until the late stage of litter decomposition [36]. However, in this experiment, the effects on the contents of lignin, cellulose, and hemicellulose were not obvious, which may be because the period of this experiment was only five months, which is too short a time to show the effect of endophytic bacteria.

### 4.3. Final Release Characteristics of Litter Lignin, Cellulose, and Hemicellulose

The release rate of cellulose in the litter of *Rhododendron delavayi* and *Rhododendron agastum* was highest after the application of exogenous substances. The final release rates of lignin, cellulose, and hemicellulose in the mixed forest were lower than those in the two single stands, which resulted in the final mass loss rate of mixed forest litter after the application of exogenous substances being lower than that of *Rhododendron delavayi* and *Rhododendron agastum*. The results are consistent with a previous study, in which the decomposition of pure-stand litter was higher than that of mixed-stand litter [37]. The decomposition of litters in mixed forests may be related to the mixing ratio of different stands of litters [38].

Therefore, from the point of the final release rate, cellulose release rates of *Rhododendron delavayi* and *Rhododendron agastum* are higher than their lignin and hemicellulose release rates, which is similar to the research results of Yue et al. on lignin and cellulose degradation in the decomposition process of litter in alpine forests [31]. The cellulose concentration of litter in the mixed forest fluctuated greatly during the decomposition period and the variation trend of each treatment was not regular. The reason may be that there were an increasing number of miscellaneous leaf species in litter in the mixed forest which influenced each other.

However, exogenous N and P to the litter of *Rhododendron delavayi* could inhibit the release of lignin and cellulose to some extent. Alkali lime and microbial agents can promote the release of lignin and cellulose. Among them, YJF had a better promotion effect on lignin release, and EM had a better promotion effect on cellulose release. Applying low concentrations of N and high concentrations of P to *Rhododendron agastum* litter can promote the release of lignin, but high concentrations of N can inhibit the release of cellulose, which is similar to the research results of high levels of N to C. chinensis leaves reducing the decomposition of cellulose [39]. The study found that nitrogen addition promoted litter decomposition, but with the increase in nitrogen application level, the promoting effect gradually decreased and the decomposition of litters was inhibited after reaching a certain concentration [40]. Some studies suggest that a high concentration of nitrogen would inhibit the activity of lignin-decomposing enzymes, thus having a negative effect on the decomposition of litter [41]. Alkali lime promoted lignin release, and HS inhibited cellulose release. The release effect of the three microbial agents on lignin and cellulose was not obvious. The release of hemicellulose increased to different degrees under different treatments, indicating that N, P, alkali lime, or microbial agents had different degrees of promoting effects on the release of hemicellulose.

## 5. Conclusions

Our study demonstrates that artificial regulation measures are effective for accelerating the decomposition of litters in rhododendron stands. In particular, low concentrations of N and P can promote the decomposition of the three types of litter. The most effective measures to accelerate the decomposition of litter of different rhododendrons are also different. For *Rhododendron delavayi* litter, YJF was the most effective. For *Rhododendron agastum* litter, LN and EM were both effective. For mixed forest litter, alkali lime addition was the most effective. The forestry sector can speed up the decomposition process of litters by applying low concentrations of N and P in future management and can also apply corresponding effective measures to a specific litter type.

## Figures and Tables

**Table 1:** Initial contents of lignin, cellulose, and hemicellulose in litters of three stands.

Forest Stands	Lignin Content/%	Cellulose Content/%	Hemicellulose Content/%
*Rhododendron delavayi*	11.41 ± 0.28 b	11.21 ± 0.07 b	3.61 ± 0.15 b
*Rhododendron agastum*	13.01 ± 0.09 a	14.24 ± 0.09 a	4.15 ± 0.49 a
Mixed forests	11.38 ± 0.25 b	5.87 ± 0.06 c	3.91 ± 0.18 ab

The different lowercase letters indicate significant differences (*p* < 0.05) among different stands.

**Table 2:** Comparison of stands and treatments on final content of lignin, cellulose, and hemicellulose.

Variable	Stands	Treatments	Stands × Treatments
Lignin final content	<0.001	<0.001	<0.001
Cellulose final content	<0.001	<0.001	<0.001
Hemicellulose final content	<0.001	<0.001	<0.001

Terms for ANOVA model (degree of freedom in parenthesis): stands (2), treatments (9), stands × treatments (18), residual (60), total (90).

## Data Availability

The data presented in this study are available on request from the corresponding author. The data are not publicly available due to privacy and sensitive nature.
